# Supplementary material for: End Sequence Analysis Toolkit (ESAT) expands the extractable information from single-cell RNA-seq data
Source: Genome Res. 2016 Oct;26(10):1397–410. doi: 10.1101/gr.207902.116 (PMC5052061; doi:10.1101/gr.207902.116)
Supplement: Supplemental Material [file supp_gr.207902.116_Supp_Materials.tar.gz › ESAT-esat_v0.1/doc/html/classumms_1_1core_1_1model_1_1score_1_1_window_all_score.html]

ESAT: umms.core.model.score.WindowAllScore Class Reference


ESAT


- Main Page
- Related Pages
- Packages
- Classes
- Files

- Class List
- Class Index
- Class Hierarchy
- Class Members

All Classes Namespaces Files Functions Variables Enumerator Pages

- umms
- core
- model
- score
- WindowAllScore

Classes |
Public Member Functions |
Static Public Attributes |
List of all members

umms.core.model.score.WindowAllScore Class Reference

Inheritance diagram for umms.core.model.score.WindowAllScore:

[legend]

Collaboration diagram for umms.core.model.score.WindowAllScore:

[legend]

|  |  |
| --- | --- |
| Classes | |
| class | **Processor** |
|  | |

|  |  |
| --- | --- |
| Public Member Functions | |
|  | WindowAllScore (Annotation a) |
|  | |
|  | WindowAllScore (AlignmentModel numerator, AlignmentModel denominator, Annotation annotation) |
|  | |
|  | WindowAllScore (AlignmentModel numerator, AlignmentModel denominator, Annotation annotation, double numeratorRegionTotal, double denominatorRegionTotal, double rapLambda, double backgroundLambda) |
|  | |
| void | setNumeratorCount (double d) |
|  | |
| void | setDenominatorCount (double d) |
|  | |
| void | setNumeratorTotal (double d) |
|  | |
| void | setDenominatorTotal (double d) |
|  | |
| void | setNumeratorRegionTotal (double d) |
|  | |
| void | setDenominatorRegionTotal (double d) |
|  | |
| double | getNumeratorCount () |
|  | |
| double | getDenominatorCount () |
|  | |
| double | getNumeratorTotal () |
|  | |
| double | getDenominatorTotal () |
|  | |
| double | getNumeratorRegionTotal () |
|  | |
| double | getDenominatorRegionTotal () |
|  | |
| double | getDenominatorLambda () |
|  | |
| double | getNumeratorLambda () |
|  | |
| double | getNumeratorRPKM () |
|  | |
| double | getDenominatorRPKM () |
|  | |
| double | getRatio () |
|  | |
| double | getScore () |
|  | |
| double | getSkellamPValue () |
|  | |
| void | setNumeratorScanPVal (double pval) |
|  | |
| void | setDenominatorScanPVal (double pval) |
|  | |
| double | getNumeratorScanPVal () |
|  | |
| double | getDenominatorScanPVal () |
|  | |
| double | getEnrichmentBinomialPValue () |
|  | |
| double | getDepletionBinomialPValue () |
|  | |
| double | getLog2Ratio () |
|  | |
| double | getRegionRatio () |
|  | |
| double | getLog2RegionRatio () |
|  | |
| double | getRegionNumeratorRPKM () |
|  | |
| double | getRegionDenominatorRPKM () |
|  | |
| String | toString () |
|  | |
| Public Member Functions inherited from umms.core.model.score.CountScore | |
|  | CountScore (Annotation a) |
|  | |
|  | CountScore (CountScore other) |
|  | |
|  | CountScore (CountScore other, Annotation newAnnotation, double newCount) |
|  | |
|  | CountScore (AnnotationCollection<?extends Annotation > model, Annotation annotation) |
|  | |
|  | CountScore (AnnotationCollection<?extends Annotation > model, Annotation annotation, double regionTotal) |
|  | |
|  | CountScore (AnnotationCollection<?extends Annotation > model, Annotation annotation, boolean fullyContained) |
|  | |
|  | CountScore (AnnotationCollection<?extends Annotation > model, Annotation annotation, double regionTotal, boolean fullyContained) |
|  | |
|  | CountScore (Annotation annotation, double count, double regionTotal, double total) |
|  | |
| double | getCount () |
|  | |
| double | getScore () |
|  | |
| double | getTotal () |
|  | |
| double | getRegionTotal () |
|  | |
| double | getRPKM () |
|  | |
| double | getRPK () |
|  | |
| void | setCount (double count) |
|  | |
| void | setTotal (double total) |
|  | |
| void | setRegionTotal (double regionTotal) |
|  | |
| String | toString () |
|  | |
| boolean | equals (Object o) |
|  | |
| int | compareTo (CountScore o) |
|  | |
| int | hashCode () |
|  | |
| void | refreshPvalue () |
|  | |
| double | getPvalue () |
|  | |
| Public Member Functions inherited from umms.core.model.score.WindowScore.AbstractWindowScore | |
|  | AbstractWindowScore (Annotation t) |
|  | |
| Annotation | getAnnotation () |
|  | |

|  |  |
| --- | --- |
| Static Public Attributes | |
| static final double | RPKM\_OFFSET = 0.001 |
|  | |
| Static Public Attributes inherited from umms.core.model.score.CountScore | |
| static double | DEFAULT\_REGION\_TOTAL = -1.0 |
|  | |

|  |  |
| --- | --- |
| Additional Inherited Members | |
| Static Public Member Functions inherited from umms.core.model.score.CountScore | |
| static double | asRPKM (double count, double total, int windowSize) |
|  | |
| Protected Attributes inherited from umms.core.model.score.WindowScore.AbstractWindowScore | |
| Annotation | annotation |
|  | |

## Constructor & Destructor Documentation

|  |  |  |  |  |  |
| --- | --- | --- | --- | --- | --- |
| umms.core.model.score.WindowAllScore.WindowAllScore | ( | Annotation | *a* | ) |  |

|  |  |  |  |
| --- | --- | --- | --- |
| umms.core.model.score.WindowAllScore.WindowAllScore | ( | AlignmentModel | *numerator*, |
|  |  | AlignmentModel | *denominator*, |
|  |  | Annotation | *annotation* |
|  | ) |  |  |

Here is the call graph for this function:

|  |  |  |  |
| --- | --- | --- | --- |
| umms.core.model.score.WindowAllScore.WindowAllScore | ( | AlignmentModel | *numerator*, |
|  |  | AlignmentModel | *denominator*, |
|  |  | Annotation | *annotation*, |
|  |  | double | *numeratorRegionTotal*, |
|  |  | double | *denominatorRegionTotal*, |
|  |  | double | *rapLambda*, |
|  |  | double | *backgroundLambda* |
|  | ) |  |  |

Here is the call graph for this function:

## Member Function Documentation

|  |  |  |  |  |
| --- | --- | --- | --- | --- |
| double umms.core.model.score.WindowAllScore.getDenominatorCount | ( |  | ) |  |

Here is the caller graph for this function:

|  |  |  |  |  |
| --- | --- | --- | --- | --- |
| double umms.core.model.score.WindowAllScore.getDenominatorLambda | ( |  | ) |  |

|  |  |  |  |  |
| --- | --- | --- | --- | --- |
| double umms.core.model.score.WindowAllScore.getDenominatorRegionTotal | ( |  | ) |  |

Here is the caller graph for this function:

|  |  |  |  |  |
| --- | --- | --- | --- | --- |
| double umms.core.model.score.WindowAllScore.getDenominatorRPKM | ( |  | ) |  |

Here is the call graph for this function:

Here is the caller graph for this function:

|  |  |  |  |  |
| --- | --- | --- | --- | --- |
| double umms.core.model.score.WindowAllScore.getDenominatorScanPVal | ( |  | ) |  |

Here is the caller graph for this function:

|  |  |  |  |  |
| --- | --- | --- | --- | --- |
| double umms.core.model.score.WindowAllScore.getDenominatorTotal | ( |  | ) |  |

Here is the caller graph for this function:

|  |  |  |  |  |
| --- | --- | --- | --- | --- |
| double umms.core.model.score.WindowAllScore.getDepletionBinomialPValue | ( |  | ) |  |

Binomial test: combine numerator and denominator counts, then calculate cumulative probability of seeing less than this proportion

Returns
:   -log10 p-value for the numerator having less reads than expected.

Here is the call graph for this function:

Here is the caller graph for this function:

|  |  |  |  |  |
| --- | --- | --- | --- | --- |
| double umms.core.model.score.WindowAllScore.getEnrichmentBinomialPValue | ( |  | ) |  |

Binomial test: combine numerator and denominator counts, then calculate cumulative probability of seeing >= this proportion

Returns
:   -log10 p-value for the numerator having more reads than expected.

Here is the call graph for this function:

Here is the caller graph for this function:

|  |  |  |  |  |
| --- | --- | --- | --- | --- |
| double umms.core.model.score.WindowAllScore.getLog2Ratio | ( |  | ) |  |

Here is the call graph for this function:

Here is the caller graph for this function:

|  |  |  |  |  |
| --- | --- | --- | --- | --- |
| double umms.core.model.score.WindowAllScore.getLog2RegionRatio | ( |  | ) |  |

Here is the call graph for this function:

|  |  |  |  |  |
| --- | --- | --- | --- | --- |
| double umms.core.model.score.WindowAllScore.getNumeratorCount | ( |  | ) |  |

Here is the call graph for this function:

Here is the caller graph for this function:

|  |  |  |  |  |
| --- | --- | --- | --- | --- |
| double umms.core.model.score.WindowAllScore.getNumeratorLambda | ( |  | ) |  |

Here is the caller graph for this function:

|  |  |  |  |  |
| --- | --- | --- | --- | --- |
| double umms.core.model.score.WindowAllScore.getNumeratorRegionTotal | ( |  | ) |  |

Here is the call graph for this function:

Here is the caller graph for this function:

|  |  |  |  |  |
| --- | --- | --- | --- | --- |
| double umms.core.model.score.WindowAllScore.getNumeratorRPKM | ( |  | ) |  |

Here is the call graph for this function:

Here is the caller graph for this function:

|  |  |  |  |  |
| --- | --- | --- | --- | --- |
| double umms.core.model.score.WindowAllScore.getNumeratorScanPVal | ( |  | ) |  |

Here is the caller graph for this function:

|  |  |  |  |  |
| --- | --- | --- | --- | --- |
| double umms.core.model.score.WindowAllScore.getNumeratorTotal | ( |  | ) |  |

Here is the call graph for this function:

Here is the caller graph for this function:

|  |  |  |  |  |
| --- | --- | --- | --- | --- |
| double umms.core.model.score.WindowAllScore.getRatio | ( |  | ) |  |

Here is the call graph for this function:

Here is the caller graph for this function:

|  |  |  |  |  |
| --- | --- | --- | --- | --- |
| double umms.core.model.score.WindowAllScore.getRegionDenominatorRPKM | ( |  | ) |  |

Here is the call graph for this function:

Here is the caller graph for this function:

|  |  |  |  |  |
| --- | --- | --- | --- | --- |
| double umms.core.model.score.WindowAllScore.getRegionNumeratorRPKM | ( |  | ) |  |

Here is the call graph for this function:

Here is the caller graph for this function:

|  |  |  |  |  |
| --- | --- | --- | --- | --- |
| double umms.core.model.score.WindowAllScore.getRegionRatio | ( |  | ) |  |

Here is the call graph for this function:

Here is the caller graph for this function:

|  |  |  |  |  |
| --- | --- | --- | --- | --- |
| double umms.core.model.score.WindowAllScore.getScore | ( |  | ) |  |

Returns the number of reads overlapping this region

Returns
:   number of reds overlapping this window Returns the "score" as defined by each subclass (e.g. count, ratio, sum of annotation scores, etc.)

Implements umms.core.model.score.WindowScore.

Here is the call graph for this function:

Here is the caller graph for this function:

|  |  |  |  |  |
| --- | --- | --- | --- | --- |
| double umms.core.model.score.WindowAllScore.getSkellamPValue | ( |  | ) |  |

Based on prussell's function in PairedSampleCoverage Compute Skellam P-value of read counts in a region given the two parameters denominatorLambda Poisson lambda for background sample numeratorLambda Poisson lambda for signal sample denominatorCount The count for background sample numeratorCount The count for signal sample

Returns
:   The probability under the null hypothesis of observing a greater difference

Exceptions
:   |  |  |
    | --- | --- |
    | IOException |  |

Here is the call graph for this function:

Here is the caller graph for this function:

|  |  |  |  |  |  |
| --- | --- | --- | --- | --- | --- |
| void umms.core.model.score.WindowAllScore.setDenominatorCount | ( | double | *d* | ) |  |

Here is the caller graph for this function:

|  |  |  |  |  |  |
| --- | --- | --- | --- | --- | --- |
| void umms.core.model.score.WindowAllScore.setDenominatorRegionTotal | ( | double | *d* | ) |  |

Here is the caller graph for this function:

|  |  |  |  |  |  |
| --- | --- | --- | --- | --- | --- |
| void umms.core.model.score.WindowAllScore.setDenominatorScanPVal | ( | double | *pval* | ) |  |

Here is the caller graph for this function:

|  |  |  |  |  |  |
| --- | --- | --- | --- | --- | --- |
| void umms.core.model.score.WindowAllScore.setDenominatorTotal | ( | double | *d* | ) |  |

Here is the caller graph for this function:

|  |  |  |  |  |  |
| --- | --- | --- | --- | --- | --- |
| void umms.core.model.score.WindowAllScore.setNumeratorCount | ( | double | *d* | ) |  |

Here is the call graph for this function:

|  |  |  |  |  |  |
| --- | --- | --- | --- | --- | --- |
| void umms.core.model.score.WindowAllScore.setNumeratorRegionTotal | ( | double | *d* | ) |  |

Here is the call graph for this function:

Here is the caller graph for this function:

|  |  |  |  |  |  |
| --- | --- | --- | --- | --- | --- |
| void umms.core.model.score.WindowAllScore.setNumeratorScanPVal | ( | double | *pval* | ) |  |

Here is the caller graph for this function:

|  |  |  |  |  |  |
| --- | --- | --- | --- | --- | --- |
| void umms.core.model.score.WindowAllScore.setNumeratorTotal | ( | double | *d* | ) |  |

Here is the call graph for this function:

|  |  |  |  |  |
| --- | --- | --- | --- | --- |
| String umms.core.model.score.WindowAllScore.toString | ( |  | ) |  |

Here is the call graph for this function:

## Member Data Documentation

final double umms.core.model.score.WindowAllScore.RPKM\_OFFSET = 0.001

static

---

The documentation for this class was generated from the following file:

- java/umms/core/model/score/WindowAllScore.java


---

Generated on Fri Aug 15 2014 15:11:14 for ESAT by  

 1.8.7
